# Supplementary material for: The Effects of Heavy Metal Pollution on Collembola in Urban Soils and Associated Recovery Using Biochar Remediation: A Review
Source: Int J Environ Res Public Health. 2023 Feb 9;20(4):3077. doi: 10.3390/ijerph20043077 (PMC9966961; doi:10.3390/ijerph20043077)
Supplement: Supplementary file 1 [file ijerph-20-03077-s001.zip › ijerph-2156932-supplementary.pdf]

SUPPLEMANTARY DOCUMENT

Table S1 Levels of Pb and Cd in urban soils of different cities in China (mg kg^{-1})

Location	Samples	Pb		Cd		References
		Mean	Range	Mean	Range	
All the cities from China		1350.51	28.6-25380.55	1.58	0.15-8.59	Luo et al.,2012
Shanghai	273	70.69±5.09	-	0.52±0.05		Shi et al., 2008
Hongkong	594	93.4±37.3	5.27-404	2.18±1.02	0.02-5.89	Xiangdong Li, Poon, & Liu, 2001
Guangzhou	40	108.55±78.28	18.50-348.40	0.495±0.491	0.028-2.408	(Lu et al., 2007)
Hangzhou	25	202.16±197.62	54.00-845.00	1.59±1.41	0.29-5.70	(Chen et al., 2005; M. Zhang & Wang, 2009)
Lishui	126	63.15±21.96	29.18-166.93	0.53±0.52	0.05-1.90	(H. Wang & Lu, 2011)
Changchun	352	35.4±26.3		0.132±0.589		(X. S. Luo et al., 2012; Z. Yang et al., 2011)

Table S2.

Number of Collembola in different ecosystem services

Country	Type of sample	Number of Collembola	References
England	Urban soil	6040	(M.T. FOUNTAIN & HOPKIN, 2004)
East-China	Paddy field	792	(M. Liu et al., 2018)
South-Sueden	Forest	11.127	(Vanhee & Devigne, 2018)

Southern-equador	Protected forest area	18737	(Sun et al., 2020)
Iztaccihua volcano,mexico	Temperate forest	24,028	(García-Gómez et al., 2009)

Table S3

Comparative summary of pyrolysis system involving biochar production

Pyrolysis system	Parent material	Main products	By-products	Temperature (°C)	Sources
Slow pyrolysis	biomass	Biochar, biogas	Bio-oil	300–550	(Zhuanxi Luo et al., 2020)
Fast pyrolysis	wheat residue	Gas	Liquid and very low biochar	500-700	(Tang, Zhu, Kookana, & Katayama, 2013)

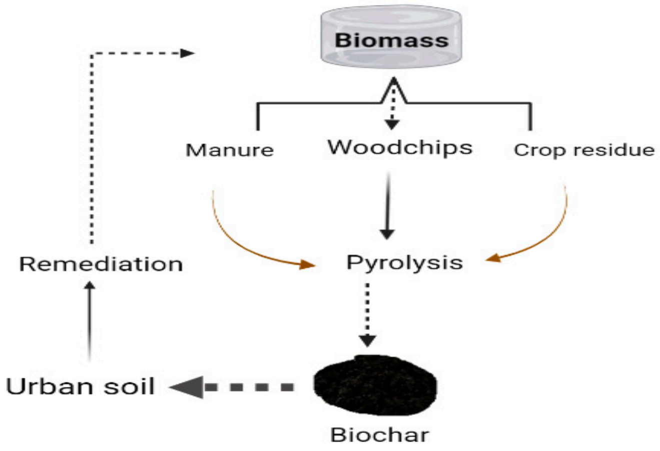

Figure S1 Summarize the process of biochar production from waste recycling, and urban soil remediation.
